# Hagfish: Champions of CO2 tolerance question the origins of vertebrate gill function

**DOI:** 10.1038/srep11182

**Published:** 2015-06-09

**Authors:** Daniel W. Baker, Brian Sardella, Jodie L. Rummer, Michael Sackville, Colin J. Brauner

**Affiliations:** 1 Department of Fisheries and Oceans, Vancouver Island University, Nanaimo, BC. Canada, V9R 5S5; 2 Department of Biological Sciences, California State University, Stanislaus, California, One University Circle, Turlock, CA, USA; 3 ARC Centre of Excellence for Coral Reef Studies, James Cook University, 1 James Cook Drive, Townsville, QLD 4811 Australia; 4 Department of Zoology, University of British Columbia, 6270 University Blvd., Vancouver, B.C., Canada, V6T 1Z4

## Abstract

The gill is widely accepted to have played a key role in the adaptive radiation of early vertebrates by supplanting the skin as the dominant site of gas exchange. However, in the most basal extant craniates, the hagfishes, gills play only a minor role in gas exchange. In contrast, we found hagfish gills to be associated with a tremendous capacity for acid-base regulation. Indeed, Pacific hagfish exposed acutely to severe sustained hypercarbia tolerated among the most severe blood acidoses ever reported (1.2 pH unit reduction) and subsequently exhibited the greatest degree of acid-base compensation ever observed in an aquatic chordate. This was accomplished through an unprecedented increase in plasma [HCO_3_^−^] (>75 mM) in exchange for [Cl^−^]. We thus propose that the first physiological function of the ancestral gill was acid-base regulation, and that the gill was later co-opted for its central role in gas exchange in more derived aquatic vertebrates.

In terrestrial vertebrates the division of gas exchange and ion/acid-base homeostasis between the lungs and kidney, respectively, is well established. In the marine environment, where vertebrate life first evolved, the vertebrate gill is used for all of these processes^1^. The gill was crucial to the adaptive radiation of early vertebrates, allowing for increased body size, activity level and skin mineralization^2^. However, the gill could not have initially evolved to satisfy all of these processes simultaneously. Indeed, it has long been held that the primary selective pressure driving early gill function was for increased O_2_ uptake, and thus gas exchange (termed the O_2_ hypothesis^2^,^3^,^4^). However, as protovertebrates increased in size and activity, the inability of the skin to maintain acid-base and ionoregulatory homeostasis may have been more limiting than that for gas exchange^4^. This appears to be the case during development in teleosts, where these processes shift from the skin to the gills long before gas exchange^5^,^6^. We thus propose that the primary selective pressure shaping early vertebrate gill evolution was for an increased acid-base relevant ionoregulatory capacity, which is supported by our findings presented here on hagfish, the most basal extant craniate (Fig. 1).

While hagfish have many derived traits, they also retain several key ancestral features deemed representative of protovertebrates^7^. Three that are particularly relevant to our hypothesis are the following: 1) they are the most basal extant deuterostome to have filamentous gills that possess a semi-permeable barrier separating blood from the external environment, 2) they have a high epidermal surface area to volume ratio (SA:V) due to a long, thin body plan (Figs. 1 and 3) they are osmo-ionoconformers. In fact, hagfish are the only extant craniates that are both osmo- and ionoconformers, with their plasma composition closely resembling seawater^8^,^9^. This is thought to represent the ancestral state because hagfish probably never invaded freshwater, and all of the more basal, extant deuterostomes (e.g., hemichordata, echinodermata) are also osmo-ionoconformers, in contrast to all of the more derived craniates^1^,^7^.

As osmo-ionoconformers, hagfish gills are not involved in ionoregulation for the purposes of ion (i.e., Na^+^ and Cl^-^) balance as in other fishes and interestingly, their gills do not appear to play much of a role in gas exchange. Instead, 80–90% of oxygen uptake in hagfishes occurs across the skin at rest^10^,^11^ due to the high epidermal SA:V characteristic of all extant or extinct early fishes (Fig. 1). Given the high dependence upon cutaneous respiration, perhaps it is not surprising that hagfish have the lowest oxygen consumption rate (*M*O_2_) of any craniate at rest or following severe stress^12^,^13^. A few studies have provided evidence for acid-base relevant ionoregulation in the hagfish gill^14^,^15^,^16^ by identifying associated gill transporters^17^,^18^,^19^ and cell types with a recent study providing the first direct support for acid extrusion *via* the gills through use of a divided chamber system^16^. However, acid-base regulatory capacity (as informed by the rate and degree of pH compensation following a disturbance) in response to an environmentally induced acidosis (as opposed to acid injection) has not been specifically investigated for comparison with other aquatic craniates.

Acid-base regulation is one of the most tightly regulated physiological processes in animals^20^. Changes in blood and cellular pH impact protein charge, and the consequences to protein function can impair everything from enzyme function, cellular ion transport, muscle contractility and metabolism through to survival^21^. While the mechanisms and capacity for acid-base regulation *via* the teleost gill have been reasonably well studied, much less is known about the hagfish gill^22^. Given the intimate association between ion and acid equivalent exchange during acid-base compensation at the teleost gill^1^ and that hagfish are ionoconformers^9^, one might expect limited acid-base compensatory capacity. Contrastingly, aspects of hagfish life history suggest otherwise: hagfish burrow in soft sediments and use their toothed tongue to enter dead animal carcasses while feeding where they may remain for extended periods^23^. These conditions promote aquatic hypoxia (low environmental O_2_) and hypercarbia (elevated environmental CO_2_), both of which can severely disrupt acid-base status. Indeed, Pacific hagfish, *Eptatretus stoutii*, are supremely tolerant of hypoxia and anoxia^13^. However, hypercarbia tolerance in hagfishes is unknown^14^, and while elevated CO_2_ is an environmentally relevant challenge, hypercarbia exposure can also be used as a tool to quantify acid-base regulatory capacity. Here we exposed hagfish to sustained hypercarbia to induce a rapid acidosis. Subsequent recovery was used to quantify the rate and degree of blood acid-base compensation for direct comparison with other aquatic craniates, which has not been previously investigated.

## Results and Discussion:

Pacific hagfish were acutely exposed to a water pCO_2_ of 10, 30 or 50 mm Hg (1.3, 4 and 6 kPa CO_2_) for up to 96 h, to simulate the most extreme conditions that hagfish could possibly experience in a benthic burrow, within a decaying carcass, in proximity to deep sea hydrothermal vents^24^, or near point sources such as CO_2_ injection sites^25^. As expected, hypercarbia triggered an immediate acidosis in Pacific hagfish that varied with the severity of hypercarbia. Within 3 h, blood pH (pHe) decreased from 7.99 (±0.02) to 7.62 (±0.01), 7.04 (±0.03) and 6.81 (±0.03) with exposure to 10, 30 and 50 mm Hg pCO_2_, respectively (Fig. 2). Tissue (muscle, heart and liver) intracellular pH (pHi) decreased in parallel with pHe (Fig. 2), but to a lesser degree, in accordance with their higher buffering capacity (Supplementary Fig. 1) relative to blood (Fig. 3). Despite the severe acidosis, among the greatest ever reported for surviving water breathers, hagfish compensated by elevating pHe as early as 6 h but always well before the end of the 96-h exposure. Compensation of pHe was both rapid and extensive, resulting in 95% (at 24 h), 70% (at 48 h), and 65% (at 96 h) recovery at 10, 30, and 50 mm Hg pCO_2_, respectively [calculated as % return of pH to pre-exposure (i.e., 0 hr) values from the 3 h value at the respective CO_2_ tension].

Blood pH recovery was associated with elevated plasma [HCO_3_^−^] (Figs. 3,4) and an equimolar reduction in plasma [Cl^−^] (Fig. 4). Net plasma HCO_3_^−^/Cl^−^ exchange during exposure to hypercarbia is the typical pattern observed in teleosts^20^, and these data imply it likely represents the basal condition. Other plasma ions were unchanged ([Na^+^], [Mg^2+^], and [Ca^2+^]; Fig. 3). The calculated net acid excretion rate for hagfish was similar to that of other fish species investigated (Supplementary Table 1), but what stands out in the physiological response to hypercarbia is the degree of pHe compensation, as well as the associated quantitative changes in plasma [HCO_3_^−^] and [Cl^−^]. No other water-breathing craniate has been reported to either tolerate ~1.2 pH blood acidosis or to recover pHe to this degree. This impressive pHe compensation during acute hypercarbia was driven entirely by an unprecedented increase in plasma [HCO_3_^−^], in exchange for [Cl^−^], which reached 78.2 (±4.5) and 75.4 (±8.2) mM during exposure to 30 and 50 mm Hg pCO_2_ (Figs. 3,4), respectively. These values are over twice the next highest plasma [HCO_3_^−^] ever reported for a water-breathing vertebrate during acute exposure to hypercarbia^20^. Typically, water-breathing fish exposed to acute (≤96 h) hypercarbia are unable to elevate blood HCO_3_^−^ beyond 25–33 mM, termed the “bicarbonate concentration threshold”^20^ (Fig. 3). In any case, the gills of hagfish appear to be an efficacious structure for acid-base regulation with a compensatory capacity that far exceeds that of any other aquatic craniate investigated to date.

We believe that the hagfish’s tremendous upper limit for blood acid-base compensation may be associated with its’ osmo-ionoconforming strategy and consequent high plasma [Cl^−^] (458 mM; Fig. 3B) providing more anions available in the blood for HCO_3_^−^exchange. Teleosts typically have plasma [Cl^−^] of 130–150 mM^1^, and during acute hypercarbia, about 17–20% of the plasma [Cl^−^] can be exchanged with HCO_3_^−^ before the bicarbonate concentration threshold is reached. Thus, complete pHe compensation during acute hypercarbia is limited to 10–16 mm Hg pCO_2_. In hagfish, the increase in plasma [HCO_3_^−^] during hypercarbia reached a value of 78.2 ± 4.5 mM (Fig. 4A), which corresponded to 17% of control plasma [Cl^−^] (Fig. 4B), a similar proportion to that observed in teleosts that are able to compensate for acute hypercarbia. We suggest therefore that the degree of pH compensation attainable in fishes during acute hypercarbia may be limited by the relative decrease in plasma Cl^−^ levels and so linked to the importance of avoiding hypochloremia. While there are few opportunities to test this hypothesis, the greater CO_2_ tolerance of elasmobranchs compared to teleosts^26^ may also be a result of higher plasma [Cl^−^], which is intermediate between that of teleosts and hagfish^1^. Lamprey, another agnathan, have similar plasma [Cl^−^] to teleosts and have a very limited ability to compensate for acute hypercarbia (R. Shartau, personal communication), indicating that the exceptional hypercarbia tolerance of hagfish is not necessarily an agnathan trait.

**Figure 1:**
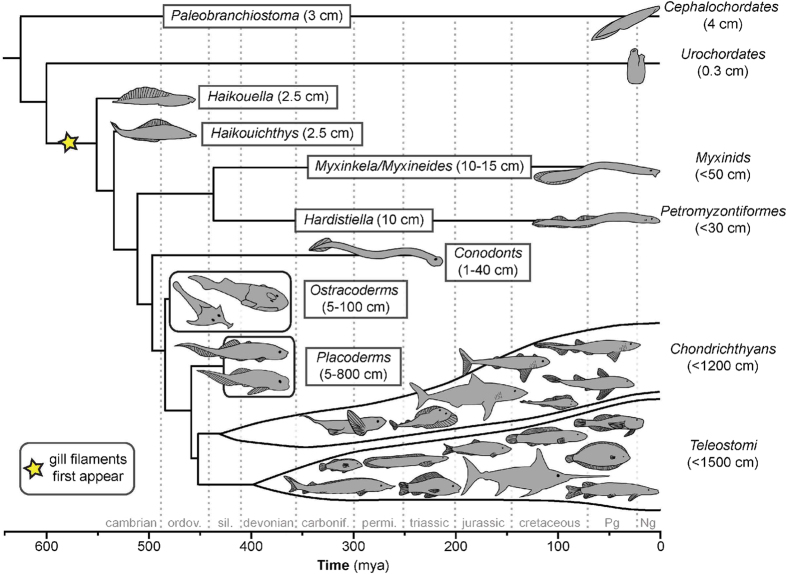
Chordate phylogeny of major extinct (boxed) and extant (unboxed) taxa (modified^34^,^35^) with maximum estimated total lengths (length data^36^). Divergence times are based on best estimates of combined molecular and fossil data^35^,^37^. The star represents the approximate appearance of gill filaments in the fossil record, which we propose were predominantly associated with acid-base regulation. Major increases in body size and/or reductions in body surface area to volume ratio occurred after this event and likely signify the transition of acid/base ion regulation from the skin to the gills.

**Figure 2:**
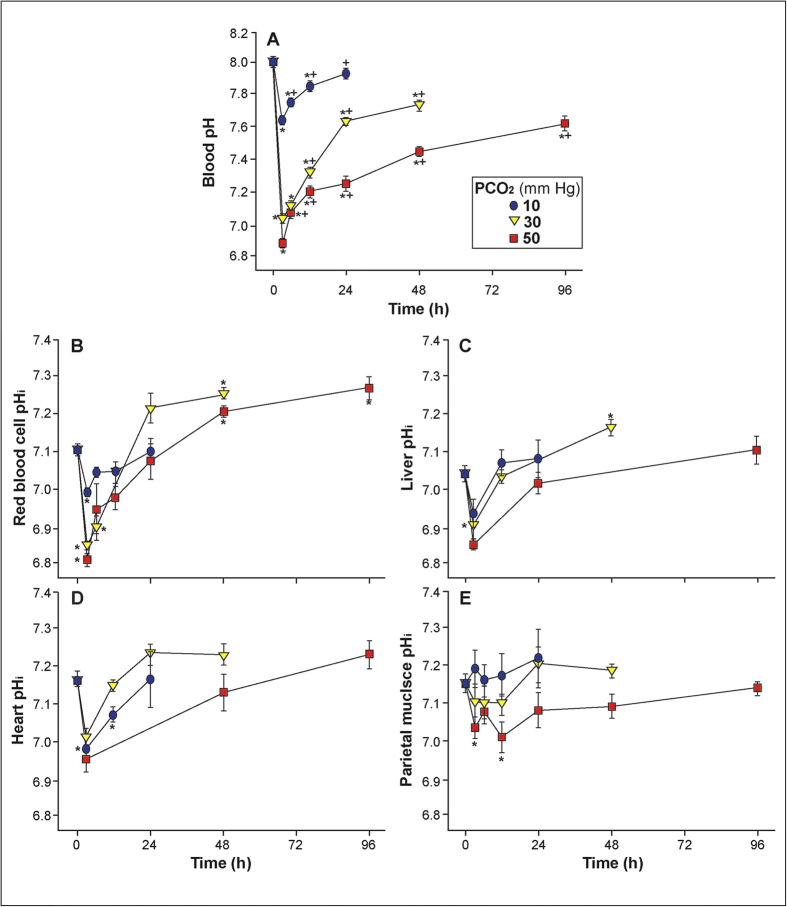
The effect of elevated water pCO_2_ (10 (•[blue]), 30 (▼[yellow]) and 50 (■[red]) mm Hg pCO_2_) for up to 96 h on A) blood pH (pHe) and intracellular pH of B) red blood cell, C) liver, D) heart and E) parietal muscle homogenates of the Pacific hagfish. All fish survived exposure up until the time of sampling. Values are means ± SEM. “*” indicates a statistically significant difference from time 0 values (p <0.05). In panel A), “+” indicates a statistically significant pH recovery from lowest pH point within a CO_2_ exposure.

**Figure 3:**
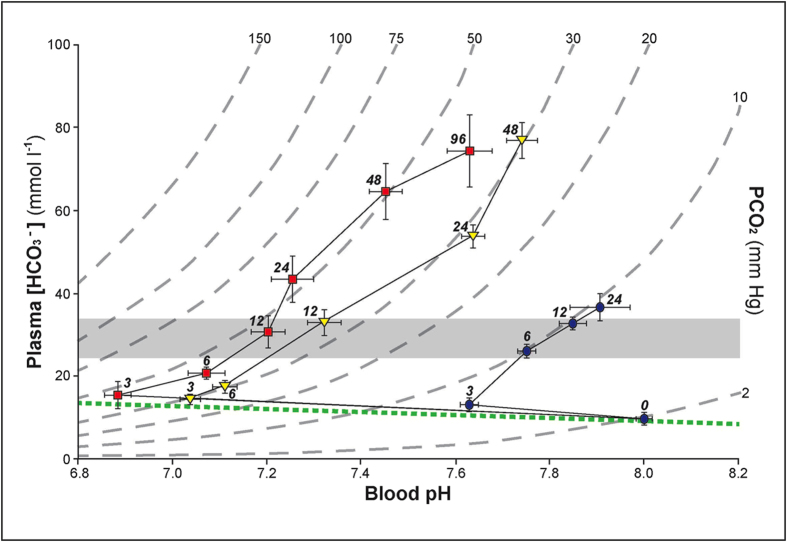
A pH/HCO_3_^−^/CO_2_ diagram of pHe compensation in Pacific hagfish during exposure to 10 (•[blue]), 30 (▼[yellow]) and 50 (■[red]) mm Hg pCO_2_. Values are means ± SEM. Curved long-dashed lines represent pCO_2_ isopleths and the short-dashed line represents the blood buffer line (5 mM [HCO_3_^−^] pH^−1^ blood). Data labels represent duration of CO_2_ exposure (**h**). Shaded area represents the “bicarbonate concentration threshold” associated with pHe compensatory limits in other fish as described previously^20^.

**Figure 4:**
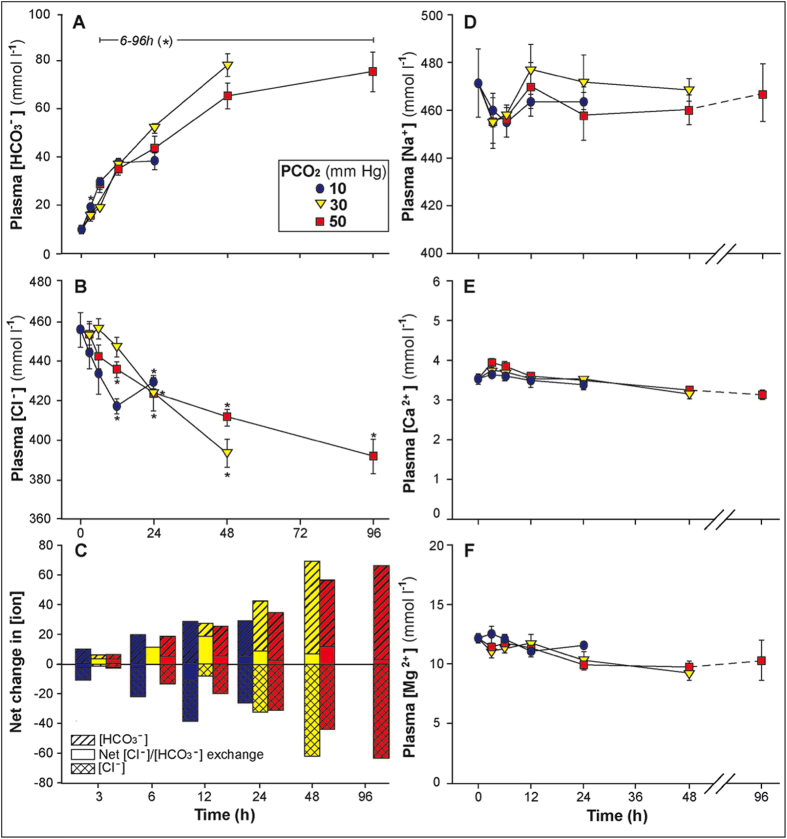
The effect of elevated water pCO_2_ (10 (•[blue]), 30 (▼[yellow]) and 50 (■[red]) mm Hg pCO_2_) on plasma **A**) [HCO_3_^−^], **B**) [Cl^−^], **D**) [Na^+^], **E**) [Ca^2+^], and **F**) [Mg ^2+^] in Pacific hagfish (mean ± SEM). In **C**), bars represent the change (relative to controls) in [HCO_3_^−^] (hatched), [Cl-] (cross hatched) and net difference between HCO_3_^−^ - Cl^−^(open) during exposure to a water pCO_2_ of 10 (blue), 30 (yellow) and 50 (red) mm Hg (approximately 1.5, 4 and 6 kPa). “*” indicates a statistically significant difference from time 0 values (p < 0.05).

In contrast, the ancestral vertebrate was likely an osmo-ionoconformer, as discussed above, where plasma [Cl^−^] may have limited the extent of acid-base compensation, and the gill dictated the rate of acid extrusion. If so, the gradual lowering of plasma [Cl^−^] associated with the evolution of ionoregulation, although leading to niche expansion and radiation into environments of varying salinity^27^,^28^, may also have reduced the ceiling on acid-base compensation. Indeed, other fishes with lower plasma [Cl^−^] that can tolerate extreme hypercarbia to levels similar to those tolerated by hagfish in this study do so using a very different strategy. The most basal actinopterygian (white sturgeon; *Acipenser transmontanus*^29^) and a few species of air breathing teleosts (marbled swamp eel; *Synbranchus marmoratus*^30^; armoured catfish; *Pterygoplichthys pardalis*^31^) can tolerate hypercarbia (~40 mmHg; 5–6 kPa pCO_2_) in the absence of pHe compensation (pHe is depressed and remains low) or active accumulation of plasma [HCO_3_^−^] and instead, preferentially and completely regulate tissue pHi^29^,^31^. Thus, preferential pHi regulation may have evolved to tolerate acid-base disturbances in the face of reduced plasma [Cl^−^] within the actinopterygiians (Fig. 1) and may be a trait associated with the evolution of air breathing in fish^31^.

Recently, there has been considerable interest in CO_2_ tolerance in marine animals, and an increased effort in estimating historical atmospheric CO_2_ levels to help predict the effects of climatic trends^25^. A few strategies have been proposed to sequester CO_2_ into deep ocean sites to reduce atmospheric “greenhouse” gases, but these methods could generate historically unprecedented local CO_2_ tensions to which deep-sea animals may be exposed^25^. In particular, there is concern that deep-sea animals may be especially sensitive to acid-base disturbances because they often have relatively low blood buffer capacity, low metabolic rates, and limited ion exchange capacity^32^,^33^. Accordingly, hagfish embody one of the most “at-risk” deep-sea animals, having among the lowest metabolic rate of any fish investigated to date^12^, relatively poorly buffered blood, and are thought to have extremely limited ion exchange capacity^9^. In contrast, this study indicates that hagfish may be among the most capable of aquatic vertebrates to cope with acid-base disturbances and tolerate high CO_2_ levels. Given that hagfishes are abundant, demersal fishes and play an important role in nutrient cycling, their exceptional CO_2_ tolerance may prove significant given some of the proposed CO_2_ disposal scenarios. It is intriguing that the most basal extant craniate, which may have remained relatively unchanged for hundreds of millions of years^7^, may turn out to be the most suited aquatic animal to survive in a high CO_2_ world.

In summary, the findings of this research indicate that the ancestral function of the vertebrate gill may have been predominantly acid-base regulation with a small role in gas exchange. From this we propose that increased capacity for acid-base regulation, rather than gas exchange, may have been the primary selective pressure shaping early evolution of the vertebrate gill. Clearly, more research on hagfish and other phylogenetically relevant animals is warranted to further test the hypothesis that the first physiological function of the ancestral vertebrate gill was acid-base relevant ionoregulation, and the gill was later co-opted for its central role in gas exchange in more derived vertebrate species.

## Methods:

Pacific hagfish (*Eptatretus stoutii*; 100–400 g) were exposed to seawater equilibrated with approximately 10, 30 and 50 mm Hg pCO_2_ and then sampled either a) immediately after transfer, time 0, or b) after 3, 6, 12, 24, 48 (only 30 and 50 mm Hg pCO_2_) or 96 (only 50 mm Hg pCO_2_) h of exposure to elevated pCO_2_. Blood was obtained from anaesthetized animals for pH, hematocrit, haemoglobin, and mean cell haemoglobin concentration (MCHC) as previously described^29^. Plasma total CO_2_ (TCO_2_), plasma ion composition, and RBC pHi were also measured^29^. Tissue pHi was measured from frozen tissues using the metabolic inhibitor method^29^. Non-bicarbonate whole blood buffer capacity and tissue non-bicarbonate buffer capacity was determined as described previously^29^, calculated from the slope of Δ[HCO_3_^−^] ΔpH^−1^, and then expressed in mmol HCO_3_^−^ pH^−1^ l^−1^ of blood or kg^−1^ of intracellular tissue water, over an *in vivo* relevant pH range. Data are presented as mean ± SEM (n = 8 in all cases except one, where n = 7). All data was analyzed for normality and equal variance before statistical analysis. Statistical differences were detected using a one-way ANOVA and, when necessary, a post-hoc Dunnett’s test. All statistical analyses were conducted using SigmaStat for Windows 3.5.0.54 (Systat Software, Inc., 2006), and all analyses were 2-tailed and interpreted using α = 0.05 to determine statistical significance.

For comparison with other species, an estimate of the increase in whole animal net acid excretion rates in hagfish exposed to hypercarbia was calculated (Supplementary Table 1) as the inverse of the net increase in whole body [HCO_3_^−^] following CO_2_ exposure relative to pre-exposure (i.e., time 0) values as has been done previously for other aquatic species^29^,^31^ (see supplemental information for details^38^,^39^,^40^,^41^,^42^,^43^,^44^,^45^,^46^,^47^,^48^). Overall, net acid excretion rates in hagfish were similar to those determined in other fish as has been observed previously^15^ (Supplementary Table 1).

## Additional Information

**How to cite this article**: Baker, D. W. *et al*. Hagfish: Champions of CO2 tolerance question the origins of vertebrate gill function. *Sci. Rep*. **5**, 11182; doi: 10.1038/srep11182 (2015).

## Supplementary Material

Supplementary Information
