# Supplementary material for: Hagfish: Champions of CO2 tolerance question the origins of vertebrate gill function
Source: Sci Rep. 2015 Jun 9;5:11182. doi: 10.1038/srep11182 (PMC4460890; doi:10.1038/srep11182)
Supplement: Supplementary Information [file srep11182-s1.pdf]

Title: Hagfish: Champions of CO_2 tolerance question the origins of vertebrate gill function

Authors: D.W. Baker*, B.A. Sardella, J.L. Rummer, M. Sackville and C.J. Brauner

Supplementary Information:

Methods:

Experimental Animals

Pacific hagfish (*Eptatretus stoutii*; 100-400g) were captured using baited traps placed at a depth of 100 meters in Barkley Sound, west of Vancouver Island, British Columbia (B.C.), Canada. These traps were transported to the Bamfield Marine Science Centre, Bamfield, B.C., Canada, where animals were transferred to opaque, covered, flow-through seawater tanks (12 °C) for 1-2 weeks prior to experiments. Hagfish were fed freshly killed salmon weekly.

Experimental Protocol

The night prior to experiments, hagfish were carefully transferred under water (to minimize stress and slime production) into 4 l clear plastic containers. Containers were transferred to a wet table, plumbed with flow-through seawater (12°C), and animals were allowed to acclimate for 24 h. Animals were not fed during this time. Following overnight acclimation, the containers were supplied with re-circulating seawater pumped from a header tank previously equilibrated with approximately 10, 30 and 50 mm Hg pCO_2 accomplished using a Cameron, Model 250 gas mixer and verified using a thermostatted (12°C) pCO_2 electrode

(E5036) displayed on a Radiometer PHM 73 (Radiometer Copenhagen). Animals remained coiled and motionless during these exposures. Animals were then sampled either a) immediately after transfer, time 0, or b) after 3, 6, 12, 24, 48 (only 30 and 50 mm Hg pCO_2) or 96 (only 50 mm Hg pCO_2) h of exposure to elevated pCO_2. To sample hagfish, the box was disconnected from the re-circulating system, and a concentrated solution of benzocaine (dissolved in 70% ethanol) was mixed into the box (final concentration 0.5 g benzocaine/L H_2O). Animals were considered to be anaesthetized when they no longer responded to external prodding (<10 min.), and a 1ml blood sample was drawn from the caudal sinus into a heparinized syringe, and placed on ice. The animal was then quickly transferred to a surgical table, decapitated and the entire heart and sections of liver and white muscle were removed and frozen in liquid nitrogen for later measurement of intracellular pH (pHi). Tissues for buffer value assessment were collected from non-CO_2 exposed animals using an otherwise similar protocol.

Blood was removed for measurement of hematocrit, haemoglobin, and mean cell haemoglobin concentration (MCHC) as previously described[29]. Blood pH was measured using a thermostated capillary electrode (BMS Mark 2, Radiometer Copenhagen) and digital pH meter (PHM 73, Radiometer Copenhagen). Another blood sample (1.5 ml) was centrifuged for 3 min. at 10000 rpm and plasma total CO_2 (TCO_2) was measured using a Corning CO_2 analyzer. The remaining packed red blood cell pellet was used to measure red blood cell pHi using the freeze thaw method[38] and the same capillary electrode as used for whole blood.

Frozen plasma and tissues were transported to the Department of Zoology, University of British Columbia where plasma samples were analysed for osmolarity (Vapor Point Osmometer, Westcor), $[Cl^-]$ (Digital Chloridometer, Radiometer), and $[Na^+]$, $[Mg^{2+}]$ and $[Ca^{2+}]$ (AA

spectrophotometer, Varion, FS240) and total protein (BSA method). Tissue pHi was measured from frozen tissues using the metabolic inhibitor method[39] validated for tissues from fish exposed to high pCO_2 tensions[40].

Non-bicarbonate whole blood buffer capacity was determined on a caudal blood sample (4 ml) maintained in an Eschweiller thermostated (12°C) glass tonometer, equilibrated for 45 min to a pCO_2 of 3.5, 7.5, 11, 21, and 45 mmHg using Sierra Mass flow controllers that mixed air and CO_2 (n=4 for each CO_2 level). At each CO_2 tension, blood was removed and TCO_2 and pH were measured as described above. Tissue non-bicarbonate buffer capacity was determined as described previously[29]. Tissue homogenates (n=4) were equilibrated at 2, 7.5, 15 and 30 mm Hg pCO_2 and sampled at each CO_2 level for pH and TCO_2. Tissue water fraction was determined for calculation of intracellular fluid based on the difference between wet mass and dry mass (following drying to constant weight), which were compared well with previously reported values. CO_2 solubility constants and pK' were calculated using previously derived equations[41]. In both cases, intrinsic non-bicarbonate buffer capacity (β_{NB}) was calculated from the slope of $\Delta[HCO_3^-]\ \Delta pH^{-1}$, and then expressed in mmol $[HCO_3^-]\ pH^{-1}\ l^{-1}$ of blood or kg^{-1} of intracellular tissue water, over an *in vivo* relevant pH range.

An estimate of the increase in whole animal net acid excretion rates in hagfish was calculated for comparison with other species (Supplementary Table 1) as the inverse of the net increase in whole body $[HCO_3^-]$ following CO_2 exposure relative to pre-exposure (i.e., time 0) values as has been done previously for other aquatic species[29,31]. The hagfish was simplified to a two compartment model consisting of 33% extracellular fluid[42]; assumed to be similar in composition to plasma) and 67% intracellular fluid (assumed to be similar in composition to

parietal muscle which makes up the largest volume of the animal). Plasma [HCO_3^-] was calculated as above and parietal [HCO_3^-] was calculated based upon previous work[29,31]. Briefly, buffer capacity and pH and HCO_3^- at normocapnia (i.e., control values) of the parietal muscle was used to calculate pH and [HCO_3^-] values during passive equilibrium of the tissue with treatment CO_2 levels. Then, pH change and HCO_3^- accumulation were calculated as the difference between these estimates and the measured pH and calculated [HCO_3^-] values at 3 and 24 h. The rate of net acid excretion during exposure to 30 and 50 mm Hg pCO_2 respectively is 1.2 and 1.4 mmol h^{-1} kg^{-1} body water at 3 h (Supplementary Table 1). Most of the increase in HCO_3^- at this time point is in the intracellular compartment. When calculated at 24 h, net acid excretion rates remained high (0.8 and 0.61 mmol h^{-1} kg^{-1} body water at 30 and 50 mm Hg pCO_2 respectively), but a much greater proportion of HCO_3^- was in the extracellular space at this time. Overall, net acid excretion rates in hagfish were similar to those determined in other fish (Supplementary Table 1).

It is important to note that this method likely underestimates net acid excretion at two levels. First, using parietal muscle as a proxy for the whole animal results in a conservative estimate of acid excretion because a) this tissue demonstrated the slowest rate of HCO_3^- accumulation and the smallest degree of pH compensation of all tissues measured, partially due to its high buffer capacity, and b) the method with which we obtained buffer capacity in muscle is known to overestimate intracellular buffering[42]. Second, we assumed that active net acid excretion began immediately after exposure to hypercarbia (i.e., time 0), which is unlikely to be the case. Thus, the high rates of net acid excretion relative to other fishes reported in Supplementary Table 1 are likely conservative and they are achieved in an animal with one of the lowest resting metabolic rates ever measured.

Statistical Analyses

Data are presented as mean ± SEM. All statistical differences were detected using a one-way ANOVA and, when necessary, a post-hoc Dunnett's test. All statistical analyses were conducted using SigmaStat for Windows 3.5.0.54 (Systat Software, Inc., 2006), and all analyses were interpreted using $\alpha = 0.05$ to determine statistical significance.

Supplementary Table 1: Summary of calculated net acid excretion rate in aquatic fishes listed approximately from most basal to most derived.

Species	Net Acid Excretion Rate (mmol h^{-1} kg^{-1})	Origin of Acidosis	Medium
*Eptatretus stoutii**†	0.9-1.4[a]	1.5-6% CO_2	SW
Eptatretus stoutii[16]†	~ 0.9[b]	acid infusion	SW
Myxine glutinosa[14]†	~ 0.85[b]	5% CO_2	SW
Myxine glutinosa[15]†	~0.6[b]	acid infusion	FW
Acipenser transmontanus[29]††	0.6-1.6[a]	1.5-6%	FW
Pterygoplichthys pardalis[31]††	0.7-1.4[a]	2-4% CO_2	FW
Petromyzon marinus[43]	~ 0.5[b]	exhaustive exercise	FW
Scyliorhinus stellaris[44]‡	~ 0.6[b]	1% CO_2	SW
Conger conger[45]	~ 0.6[b]	1% CO_2	SW
Opsanus beta[46]	~ 0.5[b]	5% CO_2	SW
Myoxocephalus octodecimspinosus[47]	~0.5[b]	acid infusion	SW
Carpus carpio[48]	~ 0.2[b]	1% CO_2	FW

*present study
† *E. stoutii* and *Myxine glutinosa* are osmo- and ionoconforming vertebrates.
‡ *S. stellaris* is an ionoregulating and osmoconforming vertebrate.
†† *Acipenser transmontanus* and *Pterygoplichthys pardalis* are preferential pHi regulators, able to protect tissue pHi while blood pH is severely depressed.
a. Estimated from values of buffering capacity and net HCO_3^- accumulation (see supplemental text).
b. Estimated from the difference between net ammonia and titratable acidity excretion rates in surrounding aquatic environment (net acid flux).

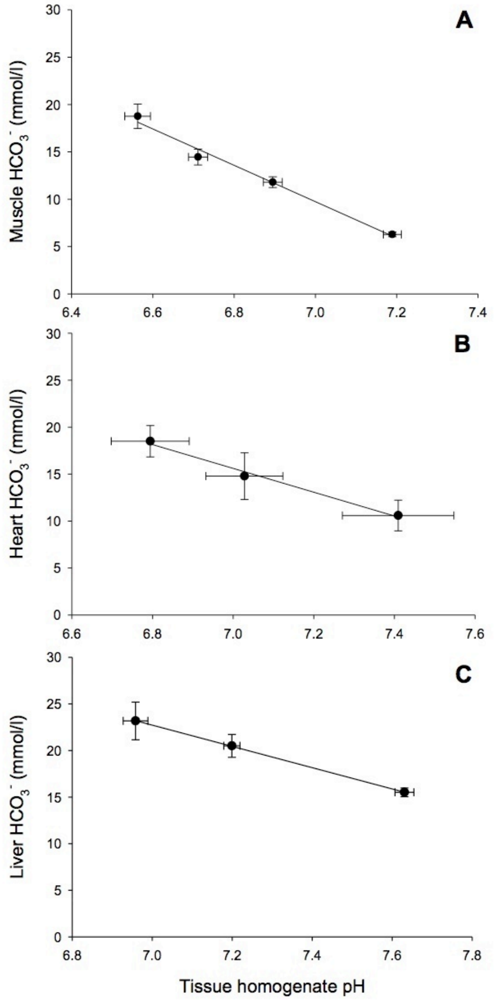

Supplementary Figure 1: The relationship between pH and [HCO_3^-] of tissue homogenates from the Pacific hagfish at different CO_2 tensions. Values are means ± SEM. Intrinsic (i.e., non-bicarbonate) buffer capacities were calculated as the following: parietal muscle = 29.3, entire heart = 22.5, and liver = 16.7 mmol HCO_3^- pH^{-1} kg^{-1} intracellular water. These values were calculated from best-fit linear regression and using estimates of extra- and intracellular tissue water for *E. stoutii* from previous work[45].
